# Case of Premature Burial

**Published:** 1913-11

**Authors:** 


Case of Premature Burial. P. A. Nightingale, M. D., District Surgeon of Victoria, S. Rhodesia, *Transvaal Med. Jour.*, June, 1913, reports an interesting case of recrudescense of an antient aboriginal custom of walling a decrepit person in a cave. A trooper of police found the old man, covered with sores and maggots, in a dying and delirious condition, buried in the regular tribal charnel house. The patient recovered. During the trial of the tribesmen who had buried him alive, it was learned that the natives believed that the spirits of phthisis, leprosy and epilepsy passed into the person who first touched the corpse of one dead of these diseases, and that, therefore, the custom was to bury such patients before death to prevent infection.

